# Insight into the Hydrolytic Selectivity of *β*-Glucosidase to Enhance the Contents of Desired Active Phytochemicals in Medicinal Plants

**DOI:** 10.1155/2018/4360252

**Published:** 2018-12-27

**Authors:** Young Soo Kim, Jin Yeul Ma

**Affiliations:** Korean Medicine Application Center, Korea Institute of Oriental Medicine, Cheomdan-ro 70, Dong-gu, Daegu 41062, Republic of Korea

## Abstract

Most glycosides in herbal medicines become pharmacologically active after hydrolysis or subsequent metabolism to respective aglycones. Hence, the hydrolytic efficiency of glycosidase is a crucial determinant of the pharmacological efficacy of herbal glycosides. In this study, we investigated the enzymatic conversion of the four herbal extracts and their glycosides using the glycoside hydrolase family 3 *β*-glucosidase from* Lactobacillus antri* (rBGLa). We show that *β*-glucosidase substrate specificity depends on the arrangements and linkage types of sugar residues in glycosides. The enzyme rBGLa showed higher hydrolytic selectivity for glucopyranoside than for glucuronide and rhamnopyranoside, and specificity for 1→6 rather than 1→2 linkages. In addition, in silico 3D structural models suggested that D243 and E426 of rBGLa act as catalytic nucleophile and acid/base residues, respectively. These experiments also suggested that substrate specificity is determined by interactions between the C6 residue of the sugar moiety of the substrate glycoside and the oxygen OD1 of D56 in rBGLa. Therefore, despite the broad substrate spectrum of *β*-glucosidase, differences in hydrolytic selectivity of *β*-glucosidases for glycoside structures could be exploited to enhance the hydrolysis of the desired medicinal glycosides in herbs using tailored *β*-glucosidases, allowing for improvement of specific potencies of herbal medicines.

## 1. Introduction

Medicinal plants often contain phenolic compounds, such as flavonoids, tannins, anthocyanins, phenolic acid, lignans, coumarins, and quinones, and are known to possess various pharmacological properties that can be exploited for various efficacies, such as anticancer, anti-inflammatory, and antioxidant effects [1–4]. Among phytochemicals, glycosides comprise backbones with various attached sugar residues, which can be efficiently extracted in hot water owing to their high aqueous solubility. Because various glycosides exert pharmacological effects after hydrolysis by intestinal microbiota and subsequent metabolism in the liver, glycoside hydrolases (GHs) of intestinal microorganisms are very important [5–9]. GHs generally catalyze hydrolytic cleavage of glycosidic bonds between the backbone and sugar residues in glycosides. Hence, to overcome differences in intestinal glycoside absorption and utilization rates caused by individual variations of intestinal microflora, attempts have been made to improve the hydrolytic efficiency of glycosides using* in vitro* bioconversion of herbal extracts with enzymes [10–12].

Based on sequence similarities and catalytic activities, GHs are classified into 153 families in EC 3.2.1 of the Carbohydrate-Active enZymes (CAZy) website. GHs may differ in their conversion efficiency with respect to specific glycosides owing to substrate specificity of enzymes caused by 3D structures even though the enzymes belong to a group with the same EC numbers. Thus, this difference in enzyme activity causes the variations in bioavailability of medicinal plants. Therefore,* in vitro* biotransformation may offer a tool for controlling the pharmacological efficacy of medicinal plants by selectively increasing the contents of desired aglycones or their metabolites based on the relationships between enzyme structures and substrate specificities.

In our previous report, we investigated the conversion of geniposide in Gardeniae Fructus to its aglycone genipin using free and immobilized recombinant *β*-glucosidase from* Lactobacillus anti* (rBGLa) [13]. However, in this study, we found that conversion rates of a glycoside hydrolase family 3 (GH3) rBGLa with EC 3.2.1.21 activity varied with glycosides in various medicinal plants, including Glycyrrhizae Radix, Gardeniae Fructus, Scutellariae Radix, and Citri Pericarpium. Thus, we investigated the effects of type of glycosides and binding structures of rBGLa on substrate specificity. Using these data, we inferred mechanisms by which rBGLa hydrolyzes glycosides in medicinal plants with* in silico* models that were based on 3D crystal structures of templates for GH3 *β*-glucosidases:* Bifidobacterium adolescentis *(BaBgl3, PDB code 5WAB),* Kluyveromyces marxianus* (KmBglI, PDB code 3ABZ), and* Thermotoga neapolitana* (TnBgl3B, PDB code 2X40) [14–16]. Finally, we analyzed functional amino acids corresponding to nucleophile and acid/base residues in the active sites of these enzymes. Through the investigation on the hydrolytic selectivity of rBGLa, we may improve target efficacy by selectively producing the desired active aglycones from medicinal plants.

## 2. Materials and Methods

### 2.1. Materials

Luria-Bertani broth, isopropyl *β*-_D_-thiogalactopyranoside, sodium phosphate (dibasic), sodium chloride, imidazole, cellobiose, and methanol were purchased from Sigma-Aldrich (St. Louis, MO, USA). Liquiritin (LQ), baicalin (BC), glycyrrhizin (GC), hesperidin (HP), neohesperidin (NH), naringin (NR), liquiritigenin, genipin, baicalein, 18*β*-glycyrrhetinic acid, hesperetin, naringenin,* p*-nitrophenol (*p*NP),* p*-nitrophenyl *β*-_D_-glucopyranoside (*p*NPG), and* p*-nitrophenyl *β*-_D_-glucuronide (*p*NPGr) standards were purchased from Sigma-Aldrich. Geniposide was purchased from Wako Pure Chemical Industries, Ltd. (Osaka, Japan), and TALON metal affinity resin was purchased from Takara (Kusatsu, Japan). Solvents for high-performance liquid chromatography (HPLC) analysis were purchased from J. T. Baker Chemical Co. (Phillipsburg, NJ, USA; acetonitrile) and Junsei Chemical Co., Ltd. (Tokyo, Japan; acetic acid).

### 2.2. Hot-Water Extraction of Medicinal Plants

Medicinal plants, including Glycyrrhizae Radix (rhizomes of* Glycyrrhiza uralensis* Fisch.), Gardeniae Fructus (dried mature fruit of* Gardenia jasminoides* Ellis), Scutellariae Radix (root of* Scutellaria baicalensis*), and Citri Pericarpium (pericarp of* Citrus unshiu* Markovich), were purchased from Yeongcheon Oriental Herbal Market (Yeongcheon, Republic of Korea) and were deposited into the herb bank of the Korean Institute of Oriental Medicine. Subsequently, 50 g samples of each medicinal plant were placed into 1,000 mL aliquots of distilled water at 115°C for 3 h (Gyeongseo Extractor Cosmos-600, Gyeongseo, Republic of Korea). After filtration through 150 *μ*m testing sieves (Retsch, Germany), extracts were freeze-dried and stored in a desiccator at 4°C. Dried powdered extracts were then stored at −20°C until use.

### 2.3. Preparation of Recombinant *β*-Glucosidase from Lactobacillus antri

rBGLa was expressed and purified using the methods described by Kim et al. [13]. In brief, the gene encoding *β*-glucosidase from* Lactobacillus antri* DSM 16041 (UniProt code: C8P9L9) was inserted into the plasmid pETDuet-1 (Novagen) between* BamH*I and* Nde*I sites by using the In-Fusion HD Cloning Kit (Takara, Kusatsu, Japan). In Luria-Bertani medium containing 50 *μ*g/mL ampicillin, cells were induced using 0.2 mM isopropyl *β*-_D_-thiogalactopyranoside for 8 h at 25°C and were then harvested by centrifugation and resuspended in washing buffer containing 50 mM sodium phosphate (dibasic) and 0.3 M sodium chloride (pH 7.0). Resuspended cells were then disrupted by ultrasonication (Omni-Ruptor 4000; Omni International, Inc., Kennesaw, GA, USA) and cell debris was eliminated by centrifugation at 20,817 x g at 4°C for 30 min. After binding of cell lysates to TALON metal affinity resins, non-specifically bound proteins were removed by washing with buffer containing 30 mM imidazole (pH 7.0). Finally, 6 × histidine-tagged rBGLa was eluted in a buffer containing 50 mM Na_2_HPO_4_ (pH 7.0), 0.3 M NaCl, and 250 mM imidazole.

### 2.4. Enzymatic Conversion of Medicinal Plants and Glycosides Using rBGLa

**Figure 1:**
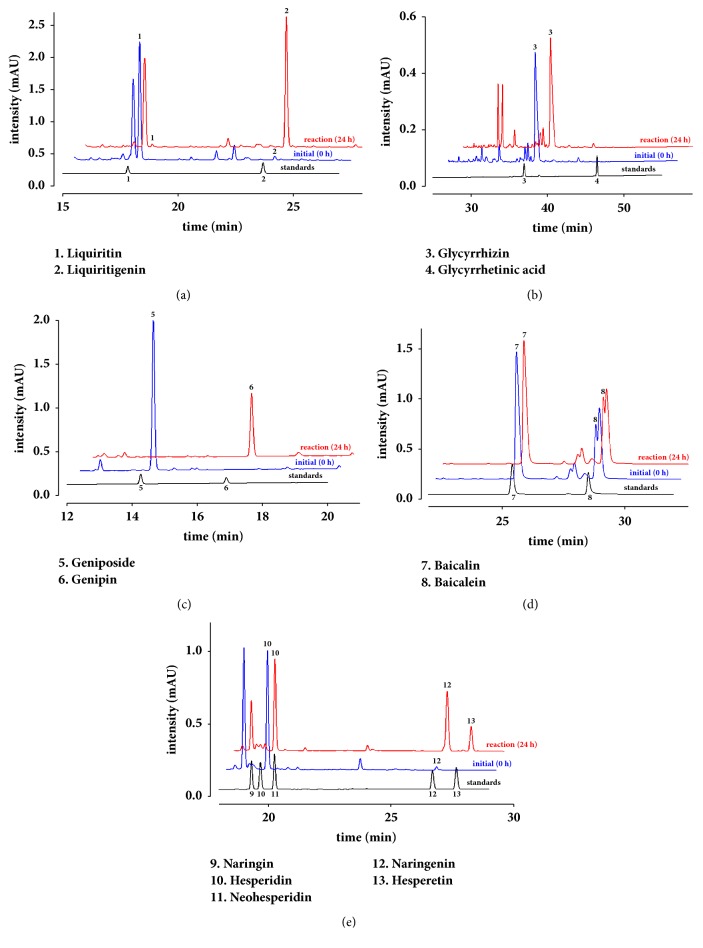
HPLC profiles for the bioconversion of four herbal extracts using recombinant *β*-glucosidase from* Lactobacillus anti* (rBGLa): Glycyrrhizae Radix at (a) 280 and (b) 254 nm, (c) Gardeniae Fructus at 240 nm, (d) Scutellariae Radix at 280 nm, and (e) Citri Pericarpium at 280 nm. Herbal extracts (20 mg/mL) were reacted with 100 *μ*g/mL rBGLa in 25 mM sodium phosphate buffer (pH 6.0) at 45°C and the samples were withdrawn at 0 and 24 h after the start of the reactions.

**Figure 2:**
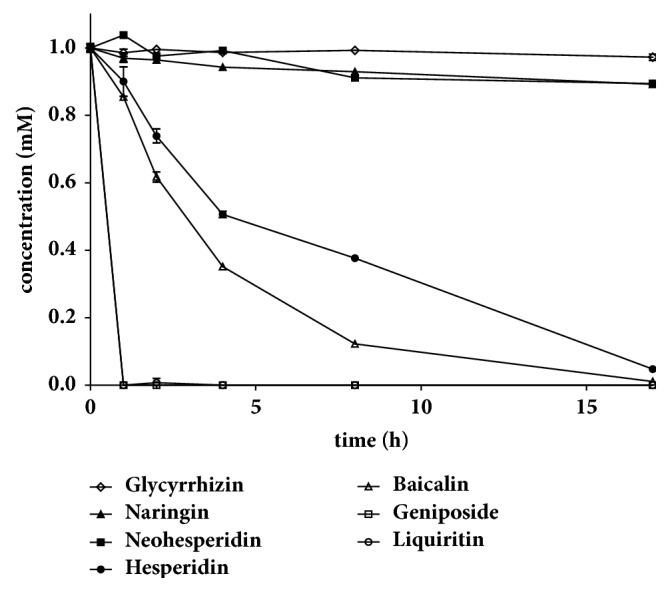
Enzymatic conversion of the glycosides liquiritin, geniposide, baicalin, glycyrrhizin, hesperidin, neohesperidin, and naringin using recombinant *β*-glucosidase from* Lactobacillus anti* (rBGLa). Each glycoside (1 mM) (pH 6.0) was incubated with 100 *μ*g/mL rBGLa at 45°C for 17 h, and the concentration of samples withdrawn at 0, 1, 2, 4, 8, and 17 h after the start of the reaction was quantified by HPLC analysis in triplicate.

For rBGLa reaction, all glycosides were first dissolved in 100% DMSO to 20 mM and diluted in reaction buffer. The diluted substrates were completely dissolved after incubation at 45°C. Then, herbal extracts (20 mg/mL) and glycosides (1 mM), dissolved in 25 mM sodium phosphate buffer (pH 6.0), were reacted with 100 *μ*g/mL rBGLa at 45°C for Figures 1 and 2. Samples containing herbal extracts were withdrawn after 0 and 24 h and those containing phytochemicals were withdrawn after 0, 1, 2, 4, 8, and 17 h. Samples were then evaporated using a speed vacuum evaporator (Modul 4080C; Hanil science medical, Daejeon, Republic of Korea) and were dissolved in methanol. Samples for biotransformation of Gardeniae Fructus and Scutellariae Radix were diluted 4-fold with methanol prior to analyzing changes in chemical compositions using HPLC.

### 2.5. Evaluation of rBGLa Activity and Kinetics with Various Glycosides

The specific activity of rBGLa enzyme was evaluated by incubating phytochemicals (1 mM) with various concentrations of rBGLa in 25 mM sodium phosphate buffer (pH 6.0) at 45°C. Reactions with liquiritin and geniposide were performed with rBGLa at 0.1 *μ*g/mL, and those with baicalin, glycyrrhizin, hesperidin, neohesperidin, and naringin were performed with 100 *μ*g/mL rBGLa. After evaporating samples that were withdrawn after reaction times of 0, 1, 2, 4, 8, and 17 h, precipitates were dissolved in methanol. The specific activities were calculated by HPLC analysis in the linear parts of the plot that displayed the amount of substrates disappeared per unit time by the rBGLa reaction. In addition, specific activities and kinetics of rBGLa at 1 and 200 *μ*g/mL were evaluated using the optimal substrates* p*NPG at 0.1-5.0 mM and* p*NPGr at 0.5–200 mM, respectively, in 25 mM sodium phosphate buffer (pH 6.0) at 45°C. Enzyme inhibition was also assessed by adding 0.1 mM cellobiose to 1 mM* p*NPG as a substrate for 1 *μ*g/mL rBGLa. During the reaction the samples were withdrawn at 2-minute intervals for 40 min (*p*NPG) and 3-minute intervals for 105 min (*p*NPGr) and liberated* p*NP concentrations were immediately measured at 405 nm using a microplate reader (GloMax-Multi Microplate Multimode Reader; Promega, Madison, WI, USA) and specific activities were calculated as *μ*mol hydrolyzed or generated/min/mg rBGLa. Km and kcat values were calculated based on the Michaelis-Menten equation (nonlinear regression) using GraphPad Prism 5.0 software.

### 2.6. Sequence Analysis and Homology Modeling of rBGLa

Three-dimensional structures of rBGLa were generated using homology modeling with SWISS-MODEL automated server (http://swissmodel.expasy.org/) based on each template (PDB codes, 5WAB, 3ABZ, and 2X40) [17], and comparative structural analyses were performed by overlapping model structures with each template using UCSF Chimera software. The protein sequence of rBGLa was also aligned with three templates using the SWISS-MODEL server. Target–template sequence identities were calculated as the ratio of the number of matched amino acids with template to the total number of amino acids and sequence similarity scores were represented as the sum of the substitution scores divided by the number of aligned residues, which were provided by SWISS-MODEL [18]

### 2.7. High-Performance Liquid Chromatography (HPLC) Analysis

HPLC (Alliance e2695; Waters Corp., Milford, MA, USA) analyses were performed with 10 *μ*L samples using a Geminin C18 column (5 *μ*m, 250 × 4.6 mm; Phenomenex Inc., Torrance, CA, USA) at an oven temperature of 40°C. The mobile phase was a gradient of acetonitrile (A) and distilled water (B) containing 1% acetic acid and was applied at a flow rate of 1 mL/min as follows: 0%–100% A (0–60 min), 100%–0% A (60–62 min), and then 0% A (62–70 min) for medicinal plants, 15%–30% A (0–5 min), 30%–15% A (5–6 min), and then 15% A (6–11 min) for GP, 28%–60% A (0–7 min), 60%–28% A (7–8 min), and then 28% A (8–13 min) for BC, LQ, HP, NH, and NR, and 60%–100% A (0–7 min), 100%–60% A (7–8 min), and then 60% A (8–13 min) for GC. Herbal extract samples were monitored under UV light at the following wavelengths: Glycyrrhizae Radix, 254 and 280 nm; Gardeniae Fructus, 240 nm; and Scutellariae Radix and Citri Pericarpium, 280 nm. Phytochemicals were also detected under UV light at 240 nm (GP), 254 nm (GC), and 280 nm (BC, LQ, HP, NH, and NR).

## 3. Results

### 3.1. Structural Analysis of Glycosides in Medicinal Plants

Medicinal plants contain multiple bioactive phytochemicals that comprise various aglycone backbones with attached sugar residues, and the structures of these sugar groups vary according to phytochemicals (Table 1). Liquorice (Glycyrrhizae Radix) contains the flavonoid liquiritin and the terpenoid glycyrrhizin, which are the major active compounds. Liquiritin has a single glucopyranoside (Glc) residue attached to the backbone liquiritigenin, whereas glycyrrhizin has a glucuronopyranosyl–glucuronide sugar group that two glucuronides (Glrs) are connected by a *β*1→2 linkage. Gardeniae Fructus contains geniposide, an iridoid glycoside linked with one Glc, as observed in licorice liquiritin. Baicalin is one of the main components of Scutellariae Radix and comprises a Glr attached to the backbone baicalein by *β*-linkage. This is a simpler sugar structure than that of glycyrrhizin. Citri Pericarpium contains hesperidin, neohesperidin, and naringin as the main active components. While hesperidin and neohesperidin have the same backbone hesperetin, the sugar residues rhamnopyranoside (Rha) and Glc are linked by *α*1→6 (rutinoside) and *α*1→2 (neohesperidoside) bonds, respectively. Furthermore, naringin has an aglycone naringenin and neohesperidoside as observed a sugar residue, as in neohesperidin.

### 3.2. Enzymatic Conversion of Medicinal Plants and Phytochemicals Using rBGLa

The extracts of medicinal plants including Glycyrrhizae Radix, Gardeniae Fructus, Scutellariae Radix, and Citri Pericarpium were biotransformed for 24 h using rBGLa, and differences in their chemical profiles were then monitored using HPLC. Liquiritin, one of the index components of Glycyrrhizae Radix, was almost completely hydrolyzed to liquiritigenin, as indicated by very low levels of the substrate in extracts after reactions (Figure 1(a)). In contrast, no concentration changes of the index component glycyrrhizin were observed (Figure 1(b)). The index component of Gardeniae Fructus, geniposide, was converted to genipin in the present rBGLa reactions that was not present in the initial extracts (Figure 1(c)). The HPLC analysis of Scutellariae Radix extract revealed the presence of the glycone baicalin and the aglycone baicalein. Although very little baicalin was hydrolyzed to baicalein, the hydrolysis of Scutellariae Radix showed a similar tendency to that observed with glycyrrhizin (Figure 1(d)). Citri Pericarpium reportedly contains the glycosides hesperidin, neohesperidin, and naringin; however, in HPLC analyses of its hot-water extract, only hesperidin was detected in large quantities, and rBGLa partially hydrolyzed hesperidin into the aglycone hesperetin with less reactivity as compared to that for liquiritin and geniposide (Figure 1(e)). In addition, increases in naringenin contents were observed after 24 h reactions with rBGLa, despite the absence of naringin in undigested Citri Pericarpium extracts.

Phytochemicals at 0.1 mM glycoside equivalent concentrations were reacted with 100 *μ*g/mL rBGLa at 45°C for 17 h at pH 6.0 (Figure 2). In these experiments, liquiritin and geniposide were completely hydrolyzed by rBGLa within 1 h, whereas baicalin and hesperidin were gradually degraded to their aglycones over 17 h, and glycyrrhizin, neohesperidin, and naringin were hydrolyzed little by rBGLa.

### 3.3. Substrate Specificity and Kinetics of rBGLa for Various Phytochemicals

To quantitatively evaluate substrate specificity, we determined and compared the enzyme activity and kinetic parameters of rBGLa with seven phytochemicals from medicinal plants (Tables 1 and 2). In these experiments, rBGLa had relatively high activity for liquiritin and geniposide, which carry Glc sugar groups, and enzyme activity for liquiritin was 16.8-fold higher than that for geniposide. Conversely, the rBGLa activity for baicalin was only 0.07% and 1.18% of those for liquiritin and geniposide, respectively, reflecting its significantly low activity for Glr residue in baicalin. In addition, rBGLa hardly hydrolyzed glycyrrhizin, in which two Glr residues are linked by a *β*1→2 bond. Consistent with these observations, rBGLa showed 81,400-fold difference in activity (*p*NPG >* p*NPGr) and significantly higher kcat and lower K_m_ values for Glc-linked* p*NPG due to the variation of sugar residues in the substrates* p*NPG and* p*NPGr, Glc and Glr. rBGLa was also found to be competitively inhibited by adding 0.1 mM cellobiose (1-*β*-_D_-glucopyranosyl-4-_D_-glucopyranose) to 1 mM substrate* p*NPG. The Km value increased 2.38-fold when compared to that for* p*NPG alone while the kcat value remained almost constant. In addition, rBGLa had significantly lower activity for the glycosides in Citri Pericarpium than for* p*NPG. Specifically, rBGLa activity was reduced to 0.02% of that for* p*NPG in the presence of hesperidin containing a rutinoside as sugar residue, in which Rha and Glc are linked by an *α*1→6 bond. Additionally, no hydrolysis of neohesperidin and naringin when bound to neohesperidoside, in which Rha and Glc are linked by an *α*1→2 bond, was observed.

### 3.4. Protein Sequence Analysis and Homology Modeling of rBGLa

**Figure 3:**
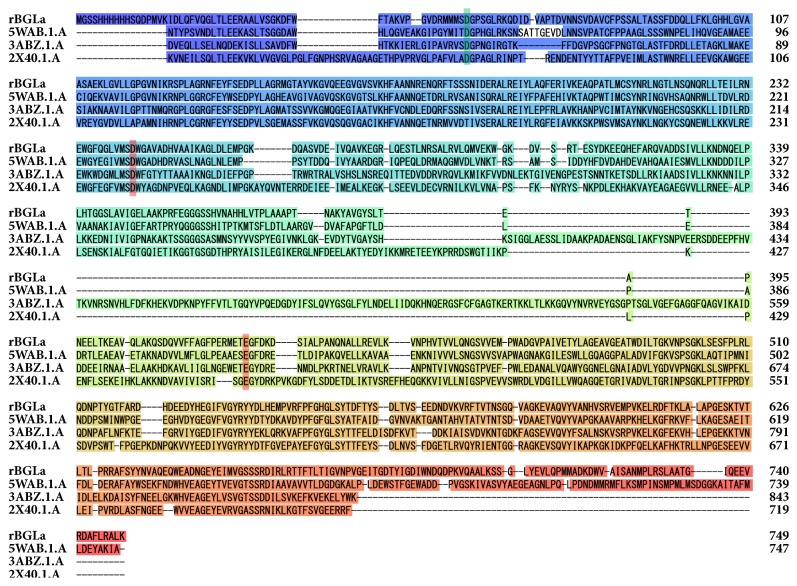
Sequence alignments of recombinant *β*-glucosidase from* Lactobacillus anti* (rBGLa) with glycoside hydrolase family 3 (GH3) *β*-glucosidase from* Bifidobacterium adolescentis *(BaBgl3, PDB code 5WAB),* Kluyveromyces marxianus* (KmBglI, PDB code 3ABZ), and* Thermotoga neapolitana* (TnBgl3B, PDB code 2X40). BaBgl3, KmBglI, and TnBgl3B were selected by global model quality estimation scores derived from modeling of rBGLa through the Swiss-Model. The functional residues predicted are indicated in red (catalytic reaction) and green (substrate specificity) boxes.

**Figure 4:**
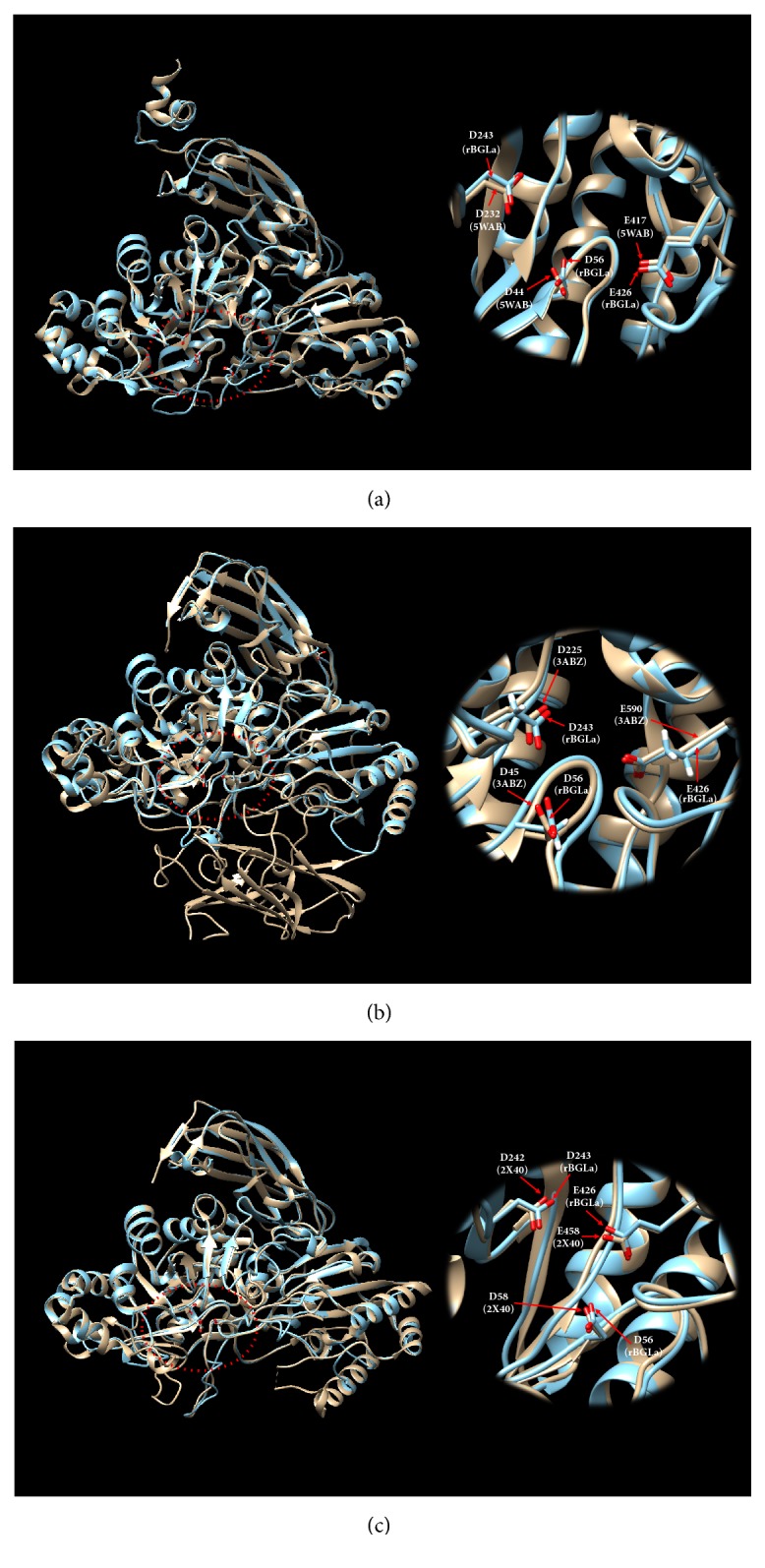
Overlap of* in silico* recombinant *β*-glucosidase from* Lactobacillus anti* (rBGLa) models with the templates (a) BaBgl3 (5WAB), (b) KmBglI (3ABZ), and (c) TnBgl3B (2X40); the 3D structure of rBGLa was built by homology modeling using the SWISS-MODEL automated server (http://swissmodel.expasy.org/) based on each template, and each model structure was overlapped with the template using UCSF chimera software.

To predict the active site of rBGLa, we built 3D structural models of rBGLa using the automatic protein homology-modeling server SWISS-MODEL based on the top three GH3 *β*-glucosidase templates:* Bifidobacterium adolescentis* (BaBgl3, PDB code 5WAB),* Kluyveromyces marxianus* (KmBglI, PDB code 3ABZ), and* Thermotoga neapolitana* (TnBgl3B, PDB code 2X40). These protein templates were selected according to global model quality estimation scores, which predict model accuracy with the score between 0 and 1, calculated based on sequence alignments and coverage scores, as follows: BaBgl3, 0.72; KmBglI, 0.67; TnBgl3B, 0.66. rBGLa had sequence similarity scores of 0.40, 0.39, and 0.38, which were provided by SWISS-MODEL based on the calculation from normalized BLOSUM62 substitution matrix [18], and identities of 42.62%, 39.78%, and 38.06% with templates of BaBgl3, 3ABZ, and TnBgl3B, respectively. The 3D structural overlaps and sequence alignments between templates and* in silico* models indicate that two Asp residues and one Glu residue are conserved in active sites comprising D56, D243, and E426 in rBGLa, D44, D232, and E417 in BaBgl3, D45, D225, and E590 in 3ABZ, and D58, D242, and E458 in TnBgl3B (Figures 3 and 4).

## 4. Discussion

Previously, we have characterized a recombinant *β*-glucosidase encoded by the genome of* L. antri *from human stomach mucosa. After expressing this in* E. coli*, we showed that this enzyme has high hydrolytic activity with geniposide, which is a glycoside from Gardeniae Fructus, and converts it to its active aglycone form genipin. Here, we investigated the conversion of sugar-linked phytochemicals in various medicinal plants to their active aglycone forms by intestinal rBGLa. Experiments with Glycyrrhizae Radix, Gardeniae Fructus, Scutellariae Radix, Citri Pericarpium, and their glycosides showed that rBGLa activities for various glycosides are subject to chemical structures of sugar residues, including arrangements and linkage types. We found that rBGLa efficiently hydrolyzed liquiritin and geniposide, which are major components of Glycyrrhizae Radix and Gardeniae Fructus, respectively. These data indicate that rBGLa selectively hydrolyzes Glcs that are linked to their backbones liquiritigenin and genipin by *β*-linkages. Despite the presence of the same sugar residues with *β*-linkages in liquiritin and geniposide, rBGLa activity for liquiritin with a flavonoid backbone was 16.8-fold higher than that for geniposide with an iridoid backbone. Hence, the backbone structure of phytochemicals significantly influences the interactions with rBGLa and the subsequent hydrolysis reactions. In contrast, baicalin, in which Glr is bound to the flavonoid backbone baicalein by a *β*-linkage, was hydrolyzed by rBGLa. Enzyme activity, however, was 1,420- and 84.3-fold lower with baicalin than with Glc-bound liquiritin and geniposide, respectively. Given these observations, we suggest that the hydrolytic selectivity of rBGLa for Glr is highly inferior to that for Glc. Accordingly, we observed an 81,400-fold difference in rBGLa activity between the substrates* p*NPG and* p*NPGr (*p*NPG >* p*NPGr), which have identical backbones but possess Glc and Glr sugar residues, respectively (Table 2). rBGLa had 55.2-fold higher affinity and 105,585-fold higher catalytic efficiency for* p*NPG than for* p*NPGr. In addition, the kinetic study also showed that rBGLa is competitively inhibited by adding 0.1 mM inhibitor cellobiose to 1 mM* p*NPG. The affinity of substrate* p*NPG to rBGLa was decreased while turnover number remained almost constant. We observed significant decreases in hydrolytic activity of rBGLa for glycyrrhizin having two consecutively bound Glr residues while* p*NPGr and baicalin possess only single Glr residues. Glycyrrhizin is absorbed into the bloodstream but is not efficacious prior to metabolism to 3*β*-monoglucuronyl 18*β*-glycyrrhetinic acid via 18*β*-glycyrrhetinic acid in the liver after hydrolysis of Glr-Glr residue [5, 7, 8]. Due to the lack of rBGLa hydrolytic activity for glycyrrhizin in Glycyrrhizae Radix, additional *β*-glucuronidase treatments may enhance the efficacy of glycyrrhizin metabolites. In addition, the significantly low hydrolytic selectivity of rBGLa for glycosides containing terminal *α*-L-rhamnose indicates that rBGLa largely fails to hydrolyze Citri Pericarpium glycosides such as naringin, hesperidin, and neohesperidin, which are linked to the backbone by Rha-Glc bonds (rutinoside and neohesperidoside). Accordingly, despite the high selectivity of rBGLa for Glc, GH3 *β*-glucosidases, such as rBGLa, cannot cleave inner *β*Glc bonds because they are exo-acting hydrolases [19, 20]. Yet, the higher activity of rBGLa for hesperidin than for naringin and neohesperidin indicates that the linkage type between two sugar residues affects the rBGLa activity, even those with the same sugar composition. Particularly, rBGLa preferred *α*1→6 linkages over *α*1→2 linkages between Rha and Glc. Previous studies also show that GH3 *β*-glucosidases predominantly hydrolyze outer Glc residues with *β*1→6 linkage at C20 of ginsenoside Rb1 rather than with *β*1→2 linkage at C3, resulting in selective production of ginsenoside Rd [21–23]. These observations suggest that GH3 family *β*-glucosidases have a higher selectivity for 1→6 linkage between sugar residues regardless of the linkage type (*α* or *β*). Similarly, rBGLa may have a much lower activity for glycyrrhizin than for baicalin due to the *β*1→2 linkage between Glr-Glr residues in glycyrrhizin.

As mentioned briefly above, the hydrolytic selectivity of GHs can be more effectively applied when converting multiple sugar residues-attached glycosides. A major ginsenoside Rb1 containing four Glcs in* Panax ginseng* can be hydrolyzed by GHs in two different metabolic pathways according to hydrolytic selectivity of GHs: Rb1→Rd→F2→Compound K (CK)→protopanaxatriol (PPT) and Rb1→Rd→Rg3→Rh2→PPT [24]. Minor ginsenoside variants produced by GHs may exhibit different pharmacological efficacies [25, 26]. The selective production of CK with anticancer effect by *β*-glucosidases [27, 28] is presumed that the *β*-glucosidases reported have the high activity of for Glc-Glc residues at the C3 position and for *β*1→2 as well as *β*1→6 linkage. In contrast, some pulsatilla saponin variants in* Pulsatilla koreana* that sugar residues can be attached at the C3, C23, and C28 positions inhibited the growth of HL-60 human leukemia cells; however, the effect of pulsatilla saponins disappeared when the sugar residues Rha(*β*1-4)Glc(*β*1-6)Glc- were attached at the C28 position [29]. In addition, pulsatilla saponins with sugar residues at the C28 position did not inhibit the inflammation in HepG2 cells [30]. Thus, this hydrolytic selectivity study is expected to be very useful to improve anticancer and anti-inflammatory efficacy of Pulsatillae Radix by selectively hydrolyzing the sugar residues at the C28 position of pulsatilla saponin.

Glycoside hydrolases, classified based on sequence similarities, are generally believed to have highly conserved 3D structures in the same class [31–33]. To further investigate the interactions between rBGLa and glycosides that may be predictive of the mechanisms of catalytic reactions, we aligned the rBGLa sequence with those of GH3 *β*-glucosidases from* B. adolescentis* (BaBgl3, PDB code 5WAB),* K. marxianus* (KmBglI, PDB code 3ABZ), and* T. neapolitana* (TnBgl3B, PDB code 2X40) and built 3D structural models using the SWISS-MODEL. Previous studies on these three templates suggested the mechanism of action of the enzymes by indicating functional amino acids, involved in electron transport during hydrolysis of glucose and interacting with sugar residues in glycosides. The amino acids D243 and E426 in rBGLa were conserved in sequence alignments with D232 and E417 in BaBglI3, D225 and E590 in KmBglI, and D242 and E458 in TnBgl3B, respectively, and these residues overlapped on 3D structures. In 3D structure analyses of BaBgl3, KmBglI, and TnBgl3B with mutant active sites, Asp appeared to act as a catalytic nucleophile residue that transfers electrons to glucose anomeric carbon, and Glu subsequently functioned as an acid/base residue to transfer electrons by forming a hydrogen bond with the *β*-anomeric hydroxyl group in the substrate [14–16]. Moreover, overlaps of* in silico* models of rBGLa with template structures indicated that the catalytic reaction follows electron transfer between D243 and E426, acting as a catalytic nucleophile and an acid/base residue, respectively. The crystal structures of BaBgL3 also revealed that the oxygen OD1 of D44 interacts with the hydroxyl group linked to C4 and C6 in glucose [14] suggesting that the oxygen OD1 of D56 is equivalent in rBGLa. Therefore, substrate specificity of rBGLa for Glc, Glr, and Rha may be due to differences in interactions between C6 residues of sugar moieties and the oxygen OD1 of D56. In agreement, we speculate that the oxygen OD1 in D56 may interact more strongly with the C6 hydroxymethyl group in Glc than with the C6 carboxyl group in Glr or the methyl group in Rha. By building the rBGLa structure through homology modeling, we could predict the key amino acids, involved in electron transfer and interacting with sugar residues in glycosides. Nevertheless, the distance and contact angle between substrates and functional amino acid residues may be changed according to the 3D coordination of amino acid residues in template and model structures, resulting in different activity and substrate specificity for the same substrate. Therefore, further investigation by the substitution of functional amino acids may reveal the 3D structure of rBGLa-activity/substrate specificity relationships. Furthermore, structural studies by homology modeling may be useful to predict hydrolytic selectivity of other glycoside hydrolases to find customized enzymes that can improve target efficacy by selectively enhancing active aglycone contents.

In this study, we focused on the selective enhancement of desired active aglycone contents based on the hydrolytic selectivity of rBGLa, which may lead to improving the increase of target efficacy. We confirmed that the activity of the GH3 family enzyme rBGLa is determined by its predominant hydrolytic selectivity to glycosides containing glucopyranoside and 1→6 linkage in sugar residues, which could enhance the contents of aglycone liquiritigenin in Glycyrrhizae Radix, genipin in Gardeniae Fructus, and hesperetin in Citri Pericarpium. The present 3D structural predictions suggested that two Asp and one Glu in rBGLa play important roles in catalytic reaction mechanisms and affinity to substrate. Thus, investigations of hydrolytic selectivity and 3D structural analysis can be used to predict the hydrolytic efficiency of glycoside hydrolases with a simple consideration of sugar residue structure of glycosides in herbal extracts. We also expect this study to be useful to convert the glycosides, to which several sugar residues are attached, such as saponins: ginsenosides in* P. ginseng* and pulsatilla saponin in* Pulsatilla koreana*. We will further investigate anti-inflammatory effects of desired minor ginsenosides, produced by the hydrolysis of major ginsenoside Rb1 and Rg1 containing four and two sugar residues, respectively, based on the hydrolytic selectivity of rBGLa

## Figures and Tables

**Table 1:** Chemical structures of glycosides in medicinal plants.

**Substrate**	**Chemical name**	**Latin name of medicinal plant**	**Structure**	**Sugar residue**	**Linkage of glycosyl group**	
					**Between sugar moieties**	**Sugar residue-backbone**
Liquiritin	Liquiritigenin-4-O-glucopyranoside	Glycyrrhizae Radix	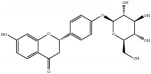	glucopyranoside	N/A^a^	*β*Glc^b^

Geniposide	Genipin-1-O-glucopyranoside	Gardeniae Fructus	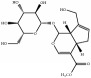	glucopyranoside	N/A	*β*Glc

Baicalin	Baicalein-7-O-glucuronide	Scutellariae Radix	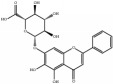	glucuronide	N/A	*β*Glr^c^

Glycyrrhizin	18*β*-glycyrrhetinic acid-3-O-glucuronopyranosyl-*β*-glucuronide	Glycyrrhizae Radix	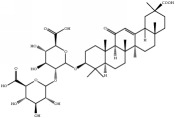	glucuronopyranosyl-glucuronide	*β*(1,2)Glr	*β*Glr

Hesperidin	Hesperetin-7-O-rutinoside	Citri Pericarpium	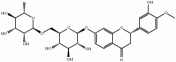	Rutinoside (rhamnopyranosyl- glucopyranoside)	*α*(1,6)Rha^d^	*β*Glc

Neohesperidin	Hesperetin-7-O-neohesperidoside	Citri Pericarpium	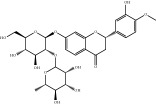	Neohesperidoside (rhamnopyranosyl- glucopyranoside)	*α*(1,2)Rha	*β*Glc

Naringin	Naringenin-7-O-neohesperidoside	Citri Pericarpium	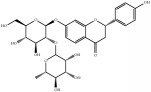	Neohesperidoside (rhamnopyranosyl- glucopyranoside)	*α*(1,2)Rha	*β*Glc

^a^N/A, not applicable; ^b^Glc, glucopyranoside; ^c^Glr, glucuronide; ^d^Rha, rhamnopyranoside.

**Table 2:** Relative specific activities and enzyme kinetics of *β*-glucosidase from *Lactobaillus antri* (rBGLa).

**Chemicals**	**Relative activity (**%**)**	**Kinetic parameter**		
		**kcat (s** ^**-1**^ **)**	**K**_**m**_ ** (mM)**	**kcat/K**_**m**_ ** (mM** ^**-1**^ ** s** ^**-1**^ **)**
*p*NPG^a^	100 ± 1	407 ± 7	1.84 ± 0.07	221 ± 9

*p*NPG + 0.1mM cellobiose	57.3 ± 1.5	432 ± 32	4.40 ± 0.57	98.1 ± 14.7

*p*NPGr^b^	0.00123 ± 0.00006	0.213 ± 0.005	102 ± 6	0.00209 ± 0.00012

Liquiritin	37.1 ± 0.3	-	-	-

Geniposide	2.20 ± 0.04	-	-	-

Baicalin	0.0261 ± 0.0002	-	-	-

Glycyrrhizin	0.000190 ± 0.000103	-	-	-

Hesperidin	0.0199 ± 0.0005	-	-	-

Neohesperidin	0.00120 ± 0.00004	-	-	-

Naringin	0.000866 ± 0.000090	-	-	-

^a^ *p*NPG, *p*-nitrophenyl *β*-_D_-glucopyranoside; ^b^*p*NPGr, *p*-nitrophenyl *β*-_D_-glucuronide.

For the measurement of relative activities, 1 mM substrates were reacted with 100 *μ*g/mL rBGLa (*p*NPG, *p*NPGr, baicalin, glycyrrhizin, hesperidin, and naringin) and 0.1 *μ*g/mL rBGLa (liquiritin and geniposide).

## Data Availability

The data used to support the findings of this study are available from the corresponding author upon request.
