# Corrigendum to “Insight into the Hydrolytic Selectivity of *β*-Glucosidase to Enhance the Contents of Desired Active Phytochemicals in Medicinal Plants”

**DOI:** 10.1155/2019/4746076

**Published:** 2019-02-25

**Authors:** Young Soo Kim, Jin Yeul Ma

**Affiliations:** Korean Medicine Application Center, Korea Institute of Oriental Medicine, Cheomdan-ro 70, Dong-gu, Daegu 41062, Republic of Korea

In the article titled “Insight into the Hydrolytic Selectivity of *β*-Glucosidase to Enhance the Contents of Desired Active Phytochemicals in Medicinal Plants” [1], the fund number K18101 should be removed. Accordingly, the Acknowledgments section should be updated as follows:

“This study was supported by Grant K17281 from the Korea Institute of Oriental Medicine, provided by the Ministry of Science and ICT, Republic of Korea.”
